# Data Collection Method for Mobile Sensor Networks Based on the Theory of Thermal Fields

**DOI:** 10.3390/s110707188

**Published:** 2011-07-14

**Authors:** Martin Macuha, Muhammad Tariq, Takuro Sato

**Affiliations:** Graduate School of Global Information and Telecommunication Studies, Waseda University, 1-3-10 Nishi-Waseda, Shinjuku-ku 169-0051, Tokyo, Japan; E-Mails: tariq@fuji.waseda.jp (M.T.); t-sato@waseda.jp (T.S.)

**Keywords:** mobile sensor networks, data collection, theory of thermal fields, human mass monitoring

## Abstract

Many sensor applications are aimed for mobile objects, where conventional routing approaches of data delivery might fail. Such applications are habitat monitoring, human probes or vehicular sensing systems. This paper targets such applications and proposes lightweight proactive distributed data collection scheme for Mobile Sensor Networks (MSN) based on the theory of thermal fields. By proper mapping, we create distribution function which allows considering characteristics of a sensor node. We show the functionality of our proposed forwarding method when adapted to the energy of sensor node. We also propose enhancement in order to maximize lifetime of the sensor nodes. We thoroughly evaluate proposed solution and discuss the tradeoffs.

## 1. Introduction

Sensor embedded systems and Wireless Sensor Networks (WSN) have attracted many researchers from academia and industry in the recent years. WSN allow many useful applications like environmental and biological monitoring [1], military surveillance [2] and infrastructure protection [3]. Usually, sensor networks are deployed for long-term monitoring, likely at the places where the exchange of the power source (battery) might not be possible and/or this replacement is very costly (e.g., underwater monitoring, leakage of harmful chemicals). Therefore, for majority of the WSN applications, data rate and delay are commonly not so important factors in the sensor networks as energy consumption and reliability.

Wireless sensor networks offer effective solutions also for vehicular sensing systems, monitoring of animal habitat and human probe systems. These applications have one common characteristic, that is mobility of sensor nodes. While vehicular systems might have sufficient resource of energy, animals (or humans) are often equipped with small monitoring tags with tiny batteries. In this paper, we would like to focus on animal and human mass monitoring applications.

One typical example of such application is monitoring of habitat in certain area like forest, national park or reservation. Depending on the information we want to periodically monitor, the frequency of data generation might vary. For behavior-related data, the data generation frequency is lower than for health-related information such as heart beat. Another very useful usage of sensors is human’s health status monitoring during mass events like concerts, large open air festivals or religious pilgrimages.

In such applications, it might be impractical (e.g., during some event) and sometimes dangerous (e.g., wildlife habitat) to change battery. A dissipated sensor node does not mean only lost of some data, but might endanger animal (human) ending by lost or death of such animal (human) or other animals. Therefore, one of the primary goals is maximizing lifetime of every node. This is in contrary with many area monitoring applications where nodes are dropped and some redundant nodes, which are sensing overlapping area, might be used for data forwarding and dissipate earlier in order to save energy of other sensor nodes located in the more important area. In habitat monitoring, every sensor node usually has the same importance in terms of lifetime, because it is monitoring a living object. Another very important aspect of this application is mobility of the sensor nodes. As animals (humans) are moving within possibly large area, it is important to reflect to the dynamicity of the network to minimize the impact on the delivery of data to the sinks. When monitoring medical or body related data, it is likely that any significant change in data should be immediately followed by reaction (e.g., medical assistance). This wireless sensor network is also relatively sensitive to end-to-end message delay between sensor node and sink.

In this paper, we propose novel data collection method which inherits the distributed and scalable nature of thermal fields and temperature transfer. The following is the summary of the key contributions:

- To the best of our knowledge, this is the first data collection method designed for mobile sensor networks based on the theory of thermal fields. In detail, we propose creation of directed acyclic graph based on the temperature, a virtual metric, which is derived from the theory of thermal fields. Then, we design lightweight proactive communication protocol for data collection, which enables forwarding data according to the warmest path.
- By using proposed algorithm we show that directed acyclic graph can be easily adapted to characteristics of node, such as energy or transmission power. We also adapt conductivity to the residual energy of the sensor node and propose Temperature exchange based Energy Adaptive Routing (TEAR) protocol for Mobile Sensor Networks (MSN).
- To maximize the lifetime of the sensor node and to prevent the node’s dissipation due to relaying, we propose minimum threshold value and self-poisoning method for sensor nodes.

In this work, we use terms “node”, “sensor node” or “sensor” interchangeably. Similarly we use terms “temperature transfer” and “heat transfer”, when referring to the same theory of thermal fields from thermodynamics. Term “temperature” refers to the virtual metric, which is inherited from the theory of thermal fields and there is no relation between this metric and real temperature of the sensor device.

The rest of this paper is organized as follows. Section 2 provides motivation behind usage of the theory of thermal fields in wireless network. Detailed description of forwarding method and mapping between the theory of the thermal fields and sensor network is presented in Section 3. Section 4 describes proposed energy adaptive protocol design. The extensive evaluation of the forwarding method and discussion is given in Section 5. Section 6 introduces major routing algorithms and relevant work for similar scenarios in MSN. Concluding comments are in Section 7.

## 2. Motivation

The theory of thermal fields describes transfer of heat between molecules in the substance and comes from thermodynamics. In this theory, a heat radiant is source of heat and emits this heat. Then, heat is transferred between molecules which are in direct contact. Each molecule will increase its temperature in dependence on neighboring molecules with higher temperatures and its own ability to transfer heat, which is called thermal conductivity. This relation is described by the second law of thermodynamics. Heat can be transferred only in one direction, from warmer molecules to the colder ones.

The one-directional transfer of heat (from warmer to the colder molecule) allows to track back to the source of heat, where the temperature reaches the maximum. This is one of the advantages of this theory, when applied in packet forwarding in such wireless networks, in which all nodes have the same destination(s). A distribution function describing this theory can be applied to WSN and the behavior of molecules could be mapped to the wireless sensor nodes. The data sink (destination) node is the source of heat with maximum (constant) temperature. Data forwarding is based on the warmest path where the warmest node of every possible path is a data sink. Thus, directed acyclic graph (DAG) is created with weights of the edges equals to the temperatures of the connected vertices over these edges.

**Figure 1.**
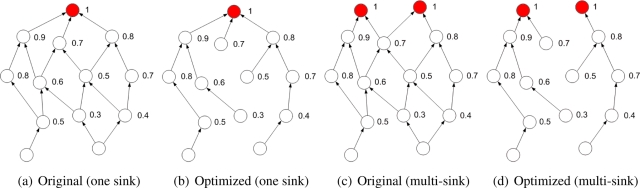
Directed acyclic graph based on heat transfer.

Simple scenario of created DAG, based on the received temperature values from direct neighbors, is shown in Figure 1(a). In Figure 1(b), the optimized DAG is shown and temperature optimization allows forwarding according to the warmest path. In Figures 1(c) and 1(d), DAG is created in the same way when two data sinks are in the network. Again, optimized DAG allows effective anycast forwarding to the neighbors with the highest temperatures when multiple sinks are in the network. The only information necessary for path selection is the temperature of the neighboring nodes.

## 3. Preliminaries

This section defines the network model, explains the theory of thermal fields and principal formulas, which are applied for DAG creation. The mapping between characteristics of node and the theory of thermal fields is also shown and different characteristics are discussed.

We assume the network consisting of sensor nodes and multiple static data sinks. All sensor nodes have limited amount of energy given by the capacity of their battery. We assume that data sinks are connected to external, unlimited, source of energy. Sensor nodes are mobile, however, there is no energy dissipation due to mobility (we assume that sensors are carried by humans/animals). Every sensor is periodically generating small amount of data (e.g., heart-beat) and this message is sent to the data sink in multi-hop manner. Thus, some sensor nodes are also involved in the relaying of messages toward the data sink. We also assume that a successful delivery of data messages to the sink does not need to be acknowledged, so there is no need for backward communication from data sink to the sensor nodes. Therefore, end-to-end communication is one-directional, the only acknowledgment is possible on the link level between two directly connected nodes.

In order to create DAG, the maximum temperature value is in the data sink, *φ_SINK_* = *MAX*, and every sensor node joining the network has initial temperature set to zero, *φ_i_* = 0. After the initial message exchange between nodes, every node calculates its own temperature based on the values of its neighbors and its own conductivity value. The temperature of node can be calculated only from neighbors, which are having higher temperature than the calculating node. Iterative algorithm for calculation of node’s temperature is shown in Algorithm 1.

**Algorithm 1.** Node *N_i_* calculates its own temperature.

*φ_i_* = 0;
sort neighbors by temperature descending;
**foreach***Neighbor N_j_* ∈ *S_N_***do**
**if***φ_i_* < *φ_j_***then**
*φ_i_* = *φ_i_* + (*φ_j_* − *φ_i_*) · *ξ_i_*;
**end**
**end**

*S_N_* is set of the node’s direct neighbors, *ξ_i_* is the thermal conductivity of node *N_i_*. *φ_i_* and *φ_j_* are the temperatures of nodes *N_i_* and *N_j_*, respectively.

### 3.1. Local Maxima Problem

To accommodate temperature transfer function in the network scenario, we must solve the problem of local maxima to ensure that forwarding by the warmest path will always end up in the data sink. When *ξ* > 1, the probability of local maxima is ≥0, therefore we modify the original function and use *ξ_NORM_* ∈ 〈0, 1). Maximum temperature is in the data sink, *φ_MAX,_* and the temperature of every non-sink node is calculated as follows:

(1) $$\varphi_{i}=\varphi_{i}+(\varphi_{j}-\varphi_{i})\cdot \xi_{i_{\mathrm{NORM}}}$$

The data sinks are thermal sources and all other sensor nodes are conducting temperature depending on their own properties. The only condition which must be kept when forwarding the packets in the network, is that there exists path from any node to any sink in the network and the temperature values of all nodes along the path are strictly increasing towards the data sink. This is accomplished by normalizing *ξ*, *ξ_NORM_* ∈ 〈0, 1) (see Theorem 1).

**Theorem 1.** *Node N_i_ is non-sink node connected to the network and there exists at least one path from node N_i_ to the sink. If ξ_NORM_* ∈ 〈0, 1) *then there exists a neighbor of N_i_, N_j_, and φ_i_* < *φ_j_*.

*Proof.* By contradiction. Assume that there is no such neighbor having *φ_i_* < *φ_j_*, so for every neighbor *N_j_*, *φ_i_* ≥ *φ_j_*. Then, from the iterative calculation of the temperature, *φ_i_*, we can write:

$$\varphi_{i}=\varphi_{i_{\mathrm{PREV}}}+(\varphi_{j}-\varphi_{i_{\mathrm{PREV}}})\cdot \xi_{i_{\mathrm{NORM}}}\wedge \varphi_{i}\geq \varphi_{j}$$

Thus

$$\begin{array}{c} \varphi_{i_{\mathrm{PREV}}}+(\varphi_{j}-\varphi_{i_{\mathrm{PREV}}})\cdot \xi_{i_{\mathrm{NORM}}}\geq \varphi_{j}\\ \xi_{\mathrm{NORM}}\geq 1 \end{array}$$

which is in contradiction with the previous assumption that *ξ_NORM_* ∈ 〈0, 1).

### 3.2. Temperature Calculation in Practice

**Figure 2.**
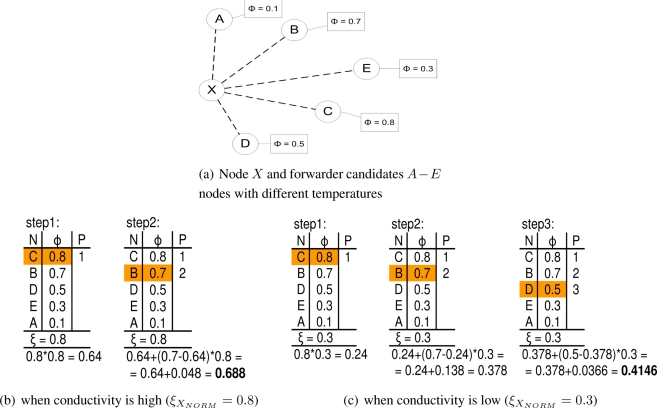
Heat computation algorithm (N-neighbor, *φ*-temperature, P-priority).

Figure 2 shows practical calculation of the node’s temperature. Assume that node *X* has neighbors *A*, *B*, *C*, *D* and *E* and knows their temperatures (*i.e.*, *φ_A_*, *φ_B_*, *φ_C_*, *φ_D_* and *φ_E_*). Then, node X creates a list of neighbors sorted descending by their temperature. Based on the Algorithm 1, the node’s temperature is calculated. From the Figures 2(b) and 2(c) is clear that node’s temperature value and also number of alternative paths varies with the node’s own thermal conductivity value *ξ_X_NORM__*. In case of high thermal conductivity value, the calculation stops after two steps and only first two neighbors (*C* and *B*) are contributing to the node’s own temperature value and only these two neighbors are potential data forwarders (column P shows priority of the next hop forwarder). In case of low thermal conductivity value, *ξ_X_NORM__* = 0.3, the node’s temperature calculation consists of three steps. The final temperature value is much lower, because of node’s low thermal conductivity value. There are three neighbors contributing to the temperature of node *X*. Thus, more alternative paths is available when one of those neighbors is disconnected. From the Equation (1) we can see that temperature of the node depends on the two factors (virtual physical attributes), namely on the temperature of the neighbors and as previously noted, on the node’s thermal conductivity value. The former one enables end-to-end path selection and the latter one depends on the characteristics of node. The characteristics of node could be mapped to node’s energy, location, importance of generated data, position in hierarchy (e.g., clusterhead), priority, transmission power or any other.

### 3.3. Distribution Function and Conductivity Value

Temperature transfer formula considers several dimensions of network in one value and we can examine its behavior by simplification. At first, this formula considers distance from the sink. This can be clearly shown by setting thermal conductivity a constant value for all sensor nodes (*ξ*_1_ = *ξ_i_* = *const*). Another dimension is the number of neighbors, thus density of the network. It can be shown that the more neighbors with the higher temperature, the higher temperature of the node itself. When conductivity value is very close to zero (*ξ* = *const* ≈ 0), the density of the network has very significant impact on the temperature value. Vice versa, if the thermal conductivity value is close to 1 (*ξ* = const ≈ 1), the distance from the data sink (number of hops) has significant impact on the temperature. Thus when the sensor nodes, which are closer to the data sink, are in good conditions (their conductivity values are close to 1), then the shortest path is preferred as the most optimal one. When those nodes are in bad conditions (their conductivity values are close to 0), more alternative paths can be found (for detailed example see description of Figure 2), thus the probability of data delivery is increased through path diversity.

This behavior can have significant impact on the network performance when thermal conductivity value is mapped to the real characteristics of node. The thermal conductivity value *ξ* can be considered as the node’s quality factor, which directly influences the temperature of node. In general, the better are the characteristics of the node, the higher is the conductivity value.

One example of mapping the thermal conductivity value to the characteristics of node is prioritization. We can assign high value (e.g., *ξ* = 0.9) to those nodes, which are preferred forwarders (high priority) and low conductivity value (e.g., *ξ* = 0.2) to the nodes which should be used for data forwarding only if necessary (low priority). Those nodes with higher thermal conductivity values are strongly influencing the temperature values of their direct neighbors, thus, are more likely the next hop forwarders for those neighbors.

Another interesting example is mapping the thermal conductivity value to the transmission power of the node. By mapping the nodes with high transmission power to high conductivity values, those nodes will be likely used as next hop forwarders. Vice versa, the nodes with low transmission power could be mapped to low conductivity values, thus, having more potential next hop forwarders (refer to Figure 2(c)). This is especially important when the node with mobility has low transmission radius (due to low transmission power) and the probability of disconnection with other nodes with mobility is high.

#### Mapping of Thermal Conductivity to Residual Energy of Node

In our proposed temperature transfer-based forwarding method we adapt the thermal conductivity value to a relative residual energy of a sensor node. The only necessary assumption is the knowledge of node’s own energy. Then the value of thermal conductivity adapted to the node’s energy can be calculated as follows:

(2) $$\xi_{E_{\mathrm{NORM}}}=\frac{E_{\mathrm{NOW}}}{E_{\mathrm{INIT}}}$$

where *E_NOW_* is current energy status (*i.e.*, remaining capacity of the battery) and *E_INIT_* is initial (maximum) energy of sensor node. Thus, when node posses full battery, the residual energy is close to 100%, thus, the thermal conductivity will be close to 1. Vice versa, when the battery is almost dissipated, the thermal conductivity of this node will be very low (close to 0).

## 4. Protocol Design

Based on the previous analysis, we propose novel Temperature exchange-based Energy Adaptive Routing (TEAR) protocol. In this section, we explain protocol design and practical issues related to the realization of the temperature transfer based communication. We split the protocol realization into two phases: Discovery phase and Forwarding phase.

### 4.1. Discovery and Forwarding Phases

Discovery phase is necessary for proper decision of the next forwarder of data. Network discovery is initialized by the sink node, which periodically sends the information about its temperature *φ_SINK_*, which is the maximum temperature and it is always constant. All neighboring sensor nodes calculate (based on the previously described Algorithm 1) own temperature and send this information to their direct neighbors. By this periodical one hop beacon exchange, the overall network is kept in the actual state. Every sensor node keeps small routing table of its neighbors with their temperature values.

Forwarding phase of temperature transfer based network is very trivial. From the previous phase, where every node calculated own temperature and created table of the neighbors, sensor node knows which neighbor is having the highest temperature. That neighbor is used for data forwarding. If the node at the first place in the routing table is disconnected, the table of neighbors is updated, the own temperature is recalculated and the warmest neighbor in the refreshed table is used for forwarding. However, in order to avoid loops, only nodes with higher temperatures than is the temperature of the actual are used for forwarding.

#### Poisoning of Weak Nodes

We also propose improvement in order to prevent the forwarding towards the node with very low energy. We define the critical conductivity value (threshold), below which the self-poisoning starts. For example, if thermal conductivity value of a node, mapped to residual energy, is *ξ_E_* < *ξ_E_MIN__*, then sensor node sets *ξ_E_* = 0 and therefore also calculated temperature of sensor node *φ* = 0. After sending the beacon with zero temperature, all neighbors having that node in their routing table as a forwarder will remove this node and it will not be used for forwarding. This node stops sending periodical beacons to save energy, however, it can still behave as a normal sensor node and send own data packets to sink as it keeps table of the neighbor nodes, which are still active (*i.e*., they are sending periodical beacons).

## 5. Results and Evaluation

We set up the network scenario similar to the application described in the first section. The simulations were performed in the Omnet++ [4] simulation tool with Energy Framework [5] developed at Technical University, Berlin. The network area is square shape with side of 1000 m and there is one data sink in every corner, totaling 4 data sinks. The sensor nodes are randomly placed into the area and are moving by Waypoint Mobility model with pedestrian speeds (uniform distribution 1–3 m/s) and pause time (uniform distribution 1–2 s). We assume using Imote2 wireless sensor node with CC2420 chipset [6]. To mitigate the energy loses from sleep mode scheduler, we assume ideal scheduler. A device is in the idle state only when it waits for message (e.g., for acknowledgments ACK) or when it performs calculation. Otherwise, device is in the sleep mode between communications (Tx or Rx modes). Ideal scheduler is unreal from the point that global knowledge or extra communication is required to make one sensor awake when another sensor node is willing to communicate with it. On the other hand, evaluation of the multi-hop forwarding methods or routing protocols might be affected by scheduler as some protocols might involve more frequent exchange of small messages, some less frequent larger message exchange, some control information exchange is periodical, some is on demand. Therefore, using one of the available schedulers for routing performance comparison could handicap one or other routing method. On the application layer, every sensor generates data messages with mean of two seconds. The simulation run lasts five minutes. When evaluating the lifetime, we run the same settings until the first node dies. Due to time and processing complexity, we set the initial battery capacity to 10 percent of the common 3 V battery capacity and then linearly extrapolated the results for lifetime of the full battery capacity. The summary of the simulation parameters is shown in the Table 1.

Based on the initial goals and the functionality of the target application, we evaluated the performance of the routing methods from the following aspects:

- *Lifetime*—time when the first sensor node completely dissipates (dies) in the system. Note, that many works refer to lifetime as the time until the last sensor node dies. As explained in the first section, our goal is to maximize lifetime of every node, not to maximize lifetime of the overall system.
- *Energetic cost*—the ratio of the total energy dissipated in the system and the number of successfully received data packets. We introduce this novel evaluation method to describe the energetic performance of the routing protocol. More closely, we are interested how much energy was spent by the system in order to deliver certain amount of the useful information (data packets).
- *Routing overhead*—number of control bits per node per second. This evaluation method shows the heaviness of the protocol and its scalability. It is also directly related to energy effectiveness as redundant control information exchange cause unnecessary dissipation of nodes. On the other hand, if there is lack of information about the network, the performance and reliability can be affected.
- *Packet delivery ratio*—the ratio of successfully delivered data packets to any data sink, and all sent data by sensor nodes. This evaluation is important as it shows how reliable is the routing method in the mobile environment.
- *End-to-end delay*—the delay from the generation of data packet in the sensor to the successful detection of the packet at the destination (data sink) at network layer. Our application assumes health related data, therefore it is relatively critical for delay. On the other hand, delay sensitivity is not of the level of multimedia transfer, therefore we believe that several seconds would not affect the overall system performance.

### 5.1. Study on the Protocol Performance

**Figure 3.**
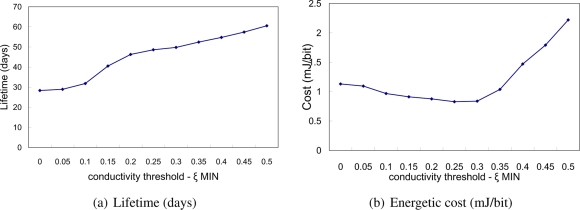
TEAR protocol performance *vs.* minimum conductivity threshold (*ξ_E_MIN__* varies from 0 to 0.5).

We used previously described simulation setting and 120 sensor nodes randomly deployed within the network area. Firstly, we investigated the proposed enhancement for TEAR protocol, *i.e.*, the minimum threshold for conductivity value mapped to the relative residual energy of the sensor node *ξ_E_MIN__*. We varied the *ξ_E_MIN__* value from 0 to 0.5 by step 0.05. When *ξ_E_MIN__* = 0, sensor node will not start own poison process. On the other hand, when *ξ_E_MIN__* = 0.5, the sensor node will start the poison process when its relative residual energy drops below 50%. Figure 3(a) shows the performance of TEAR protocol.

Clearly, the lifetime grows with increasing minimum thermal conductivity threshold *ξ_E_M_IN_*, when mapped to the energy. The self-poisoning behavior makes sensor node behaving selfishly from the point that the poisoned sensor nodes cannot be used as a forwarder afterwards. Thus, after some time, many sensor nodes are poisoned and the network becomes disconnected at the network level. To model the behavior of the sensor nodes, we investigated the energetic cost of the protocol (shown in Figure 3(b)). In this case, we simulated every run until the first sensor node dissipates and calculated the ratio between spent energy by the system and delivered useful data. It can be seen that for large *ξ_E_M_IN_* the cost is very high and has growing tendency. This is because the amount of delivered data is decreasing when many nodes cannot be used as forwarders. The cost function decreases for low *ξ_E_M_IN_* and reaches minimum when *ξ_E_M_IN_* ≈ 0.25. This is because some sensor nodes started poison process and stopped sending periodical updates (beacons) to its neighbors to save energy while there are still alternative paths for data delivery.

From the previous evaluations we found out that in order to increase lifetime of the sensor nodes, the minimum conductivity threshold value should be properly chosen. Based on our results, the sensor node should start poison process when it has remaining only 25 percent of the relative residual energy (*ξ_E_M_IN_* = 0.25).

### 5.2. Comparative Evaluation

We compared our protocol with two other protocols designed for mobile sensor networks. AODVjr protocol is from the group of MANET protocols, which have been modified for sensor networks. Another protocol is the Epidemic routing, which is aimed for mobile sensor networks.

We implemented AODVjr, a simplified version of AODV protocol, which is aimed for sensor networks. It was originally proposed in [7] and we added support for multiple sinks according to [8], which is another modification of AODV protocol for anycast routing. AODV protocol belongs into the group of reactive protocols, thus when a node does not know the route to the destination, it broadcasts route request message and waits for reply with the route towards the destination.

Epidemic routing is protocol for mobile sensor networks. We implemented this protocol according to a protocol design description in [9]. Epidemic protocol is based on the exchange of message vectors between newly connected neighbors and then, based on the requirements, the sensor nodes exchange required messages, which they do not have in their buffers. The performance of this protocol depends on the buffer size for messages (it stores also messages of other sensor nodes) and on the repetition of the anti-entropy period, which defines how often two connected neighbors exchange their updated message vectors. Based on the results in [9], we set the buffer size of sensor node to 100 messages and re-initiation of the anti-entropy period to 10 seconds.

We implemented our proposed TEAR protocol based on the previously described design and we set the period for beacon (message carrying temperature value) to 1 second. Every sensor stores table of the actually connected neighbors with their temperatures and a time when the most recent beacon came.

### 5.3. Results of Comparative Evaluation

We performed extensive simulations based on the previously described settings (see Table 1). We investigated the scalability of the protocols by varying network density from 80 sensor nodes to 240 sensor nodes within the network area.

**Figure 4.**
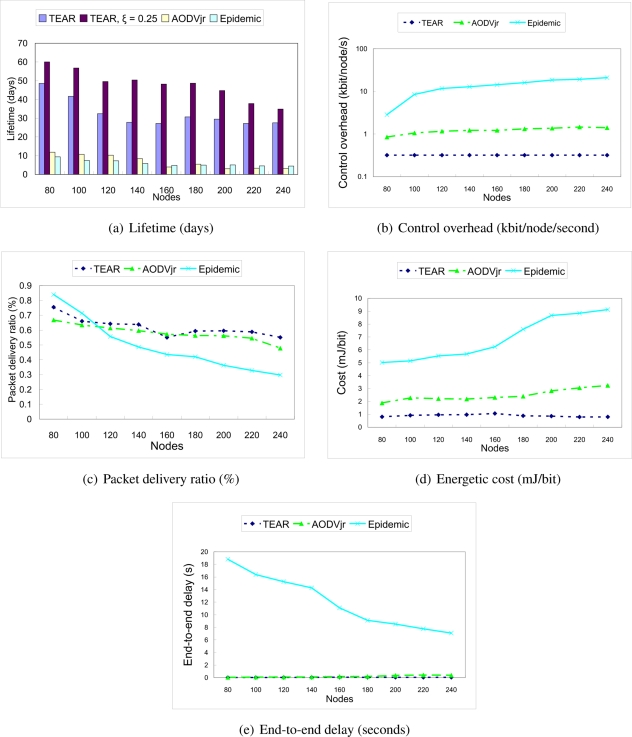
Protocol performance comparison *vs.* different density of nodes.

In Figure 4(a), we investigated lifetime of the sensor nodes. As defined earlier, we were interested in the dissipation of the first node, not the overall network. In this evaluation, we also show the performance of TEAR protocol with *ξ_E_MIN__* = 0.25. TEAR protocol has more than 4 times higher lifetime of the sensor node than Epidemic or AODVjr protocols in all cases, and when *ξ_E_MIN__* = 0.25, it has more than 5 times higher lifetime. Moreover, the lifetime of the TEAR protocol increased by more than 45% in average, when poison process is used.

Figure 4(b) shows control overhead on logarithmic scale. Epidemic protocol completely fails, causing significant control overhead in dense network (when 240 sensor nodes, control overhead is 65 times higher than TEAR protocol). Epidemic routing protocol exchanges message vectors when new sensor node is connected. Denser network increases probability of new connection, thus the sensor node performs exchange of message vectors more often. Moreover, all nodes are generating messages, thus every sensor node has many messages buffered. Therefore, the vector containing all buffered message identifiers is of very large size, causing overhead. Control overhead of AODVjr protocol also grows with the growing density, reaching 4 times higher values than TEAR protocol. This reflects to the issue of flooding. AODVjr protocol uses network flooding technique when requesting the route towards the data sink. TEAR protocol does not cause extra overhead in dense network.

In Figure 4(c), we compared packet delivery ratio of TEAR protocol, AODVjr and Epidemic protocol. In low density scenario, the Epidemic protocol overcomes other protocols as it stores the messages and transmits when connection becomes available. As the density of the network increases, the performance of the Epidemic protocol degrades significantly. This is caused by the buffer size. As the network density increases and all the sensor nodes are generating data, the buffer of the sensor node overflows, thus many data packets are dropped. TEAR and AODVjr protocols are fairly robust in the terms of the network density.

Poor performance of the Epidemic routing protocol in dense networks is reflected in Figure 4(d), spent energy per useful transferred data bit grows with the density. Energetic cost of AODVjr protocol slightly increase with the network density. This result is influenced by higher energy dissipation of the sensor nodes due to the previously described flooding issue resulting in high control overhead. For network with 240 sensor nodes, the AODV protocol has more than 4 times higher energetic cost than TEAR protocol.

Finally, end-to-end delay is compared in Figure 4(e). Epidemic protocol reaches very high delay by its nature. This delay is decreasing due to more frequent message exchange in dense network. AODVjr protocol has almost the same average delay as TEAR protocol when 80 sensor nodes in the network, however, it has 8 times higher end-to-end delay in very dense network (240 sensor nodes).

### 5.4. Discussion

From the performance of the TEAR protocol is obvious, that the exchange of temperatures between direct neighbors makes this solution very scalable and the protocol performs well regardless of the network density. Another important outcome is that the temperature is calculated without knowledge of the actual data sink. That is, the forwarding is based on anycast paradigm and if there are several data sinks in the network, the actual destination (data sink) depends on the trade-off between the distance in hops, the density towards the sink and actual residual energy of each sensor node on the path towards the data sink. Proactive behavior of our protocol design is very suitable for applications with frequent data exchange and might be ineffective in the network where only few nodes generate data or the data are generated very rarely. Another limitation of our protocol is convergence time of DAG, which depends on the period of the update message. For example, when there is sudden disconnection of data sink due to mobility of the sensor node, the temperature values of the neighbors of that data sink will drop significantly. Similarly, the neighbors of those nodes will recalculate their temperatures when received new beacon. This process will continue until all nodes with temperatures affected by that data sink update their tables and recalculate new temperature values. The overall convergence time strongly depends on the update period. In such situation, the packets are forwarded based on the outdated path, which might lead to longer paths or even packet drops due to temporal local maximum. Therefore, it is very important to properly select the update period in order to prevent temporal packet drops due to outdated paths caused by longer DAG convergence time.

## 6. Related Work

Data collection and forwarding methods in WSN have been widely studied from different perspectives, mostly focused on energy savings and network lifetime maximization. Those typical examples are well known protocols such as Low-Energy Adaptive Clustering Hierarchy (LEACH) [10], Threshold sensitive Energy Efficient sensor Network (TEEN) protocol [11], Power-Efficient Gathering in Sensor Information Systems (PEGASIS) [12] and Directed Diffusion [13]. All these protocol are aimed for static, stable and always connected networks. In our scenario, such protocols would suffer from dynamicity of the network (e.g., LEACH) or it would create huge redundancy and latency by data centric approach (e.g., Directed Diffusion), which is not appropriate method for periodical monitoring of one kind of information. Related work for our research study can be separated into two major parts, where the former one deals with routing methods in Mobile Sensor Networks and latter one is aimed on use of heat transfer in the area of wireless communications.

### 6.1. Routing in Mobile Sensor Networks

There are many works related to data harvesting in MSN, however, mostly focused on delay or fault-tolerant MSN (e.g., [14,15]).

Pervasive information gathering in Mobile Sensor Networks is studied in [14]. This human oriented data collection is analyzed when flooding and optimized flooding is used. Then, new method based on two components, namely the probability of message delivery and queue management, is proposed. Data is forwarded to the neighbor only when the neighbor has higher probability of delivery. Queue management algorithm evaluates whether to drop or transmit the message based on the fault tolerance. Another interesting solution for dynamic networks with mobile sensor nodes is epidemic routing [9]. Message delivery is ensured by random pair-wise exchange of messages among mobile sensor nodes. There are three main goals for epidemic routing, such as maximizing packet delivery, minimizing message latency and also minimizing total resources consumed in message delivery. Semi-epidemic approach is explored in [16], where the probability of replication is proportional to the time of the last encounters and their frequency. Each sensor node maintains delivery predictability vector, which indicates likelihood to meet other sensors. Data collection is based on the message forwarding from the lower predictability sensor nodes to the higher predictability sensors.

Another group of communication protocols for wireless sensor networks is based on widely studied area of wireless *ad-hoc* networks. Robustness of the routing when nodes have mobility is one of the key goals of Mobile *Ad-Hoc* Network (MANET) protocols. However, limited energy, low computation capability and memory makes direct use of such protocols difficult, sometimes prohibitive. In the recent years, several MANET protocols have been simplified and adapted to sensor networks to be more suitable for energy constraint nodes with low computational capability (e.g., [7,17,18]). *Ad-Hoc* On Demand Vector (AODV) [19] routing protocol was adapted to sensor node’s capabilities in [7] as AODVjr. The authors show that AODVjr can reach almost the same performance as the original AODV while having much less complexity. In [18], the authors proposed Not-So-Tiny AODV (NST-AODV) protocol, which provides support for mesh sensor networks. The drawback is that it requires large memory storage.

### 6.2. Theory of Thermal Fields in Wireless Networks

Recently, the theory of thermal fields and other similar strategies (gradient based or scalar field routing) has been applied to wireless networks (e.g., [13,20,21]).

In [20], a potential fields’ strategy is proposed, allowing routing based on scalar value to extreme, which can be in anycast group member. It considered as the routing decision parameter density of anycast group members in the network. Anycast group consists of multiple members and every member contributes to the field of the group. Thus, the potential field of an anycast group is defined as the superposition of the potential fields of all members in this group. The packets are routed along the steepest gradient of the potential field. The steepest gradient at each node is determined by evaluating the potential values of the neighbors. That is, the link from a node to the neighbor with the highest potential value corresponds to the steepest gradient. Therefore, the nodes always compare the potential value of their neighbors and forward anycast packets to the neighbor with the highest potential value.

In another field-based protocol, HEAT [21], the similar theory is applied to wireless mesh networks, however, the temperature of node is calculated from the density of all hosts in the network. The more neighbors with higher temperature, the higher is temperature value of the forwarding node. This approach can improve the probability of packet delivery in large mesh networks. The probability is increased by routing to denser area with anycast members instead of direct routing to the closest anycast member, because in dynamic network with frequent changes, the anycast member may become unreachable by moving out of range. The drawback of HEAT protocol is that its performance depends on the pre-set heat conductivity value, which makes trade-off between density and proximity routing. Thus, it is impractical to set the value ahead, because real network might likely have areas where proximity is preferred and other parts where density might be more suitable.

## 7. Conclusions

Heat transfer based distribution function is very promising alternative for forwarding decision in WSN. It allows to adapt the different characteristics of sensor node into one scalar value. This makes heat transfer-based forwarding specially scalable in large and also dense networks. We adapted the heat conductivity value to relative residual energy of the sensor node, designed and implemented energy-adaptive protocol and evaluated its performance. We performed extensive study on evaluation of this protocol and comparison with AODVjr and Epidemic routing protocols. While epidemic routing might be suitable for low density networks and AODVjr protocol for less dynamic networks with lower traffic, it was shown that our proposed protocol performs better in all aspects in dense networks.

We believe that this scalable technique could be used in combination with intelligent aggregation and clustering for large and dense sensor networks in mobile environment.

## Figures and Tables

**Table 1.** Simulation matrix.

Playground	1,000 × 1,000 m
Number of sensor nodes	80–240
Data sinks	4 (in the corners)
Mobility	Waypoint Mobility Model
Speed	uniform(1,3) m/s
Propagation model	Two Ray Ground Model
Radio&MAC	IEEE 802.15.4
Battery voltage	3 V
Battery capacity	1,150 mAhr
Battery capacity simulated	115 mAhr (10%)
Sleep mode	0.39 mA
Active mode, radio off	31 mA
Active mode, Tx/Rx mode	44 mA
Data packet size	512 bytes
Packet interval	*μ* = 2 s, *σ* = 0.1
Routing methods	TEAR, AODVjr, Epidemic
